# Genome editing in plants via designed zinc finger nucleases

**DOI:** 10.1007/s11627-015-9663-3

**Published:** 2015-01-29

**Authors:** Joseph F. Petolino

**Affiliations:** Dow AgroSciences, 9330 Zionsville Rd., Indianapolis, IN USA

**Keywords:** Designed nucleases, DNA repair, Gene targeting, Site-specific transgene integration, Targeted mutagenesis

## Abstract

The ability to create DNA double-strand breaks (DSBs) at specified genomic locations, which then stimulate the cell’s naturally occurring DNA repair processes, has introduced intriguing possibilities for genetic modification. Zinc finger nucleases (ZFNs) are designed restriction enzymes consisting of a nonspecific cleavage domain fused to sequence-specific DNA binding domains. ZFN-mediated DSB formation at endogenous genomic loci followed by error-prone non-homologous end joining (NHEJ) repair can result in gene-specific mutations *via* nucleotide base pair insertions or deletions. Similarly, specific DNA sequence modifications can be made by providing donor DNA templates homologous to sequences flanking the cleavage site *via* homology-directed repair (HDR). Targeted deletions of intervening DNA sequence can be obtained by ZFNs used to create concurrent DSBs. Site-specific transgene integration into ZFN-induced DSBs is possible *via* either NHEJ or HDR. Genome editing can be used to enhance our basic understanding of plant gene function as well as modify and improve crop plants. As with conventional plant transformation technology, the efficiency of genome editing is absolutely dependent on the ability to initiate, maintain, and regenerate plant cell and tissue cultures.

## Introduction

All plant traits result from complex arrays of biochemical, physiological, and developmental processes culminating in phenotypes. These processes are largely dictated by the sequences of nucleotide bases in the nuclear, plastid, and mitochondrial genomes that provide both compositional and regulatory instructions to the living cell and, consequently, the growing organism. Indeed, it is the modifications within, and recombinations among, these DNA sequences that are at the foundation of the phenotypic variability observed among organisms. By taking advantage of naturally occurring and/or induced sequence modifications and recombinations, plant breeders have made, and continue to make, substantial progress with respect to enhancing the quality and performance of crops for agricultural and industrial applications.

The last several years have seen remarkable progress in plant cell and molecular biology. Entire genome sequences have been elucidated and annotated (Michael and Jackson 2013), while databases with information on a multitude of genes and their expression have been assembled (Wingender *et al.* 2000). In addition to a deeper understanding of the dynamics of genome structure and function, modern biotechnology has provided tools that allow for the controlled alteration of DNA sequences within plant genomes. One such tool, the designed zinc finger nuclease (ZFN), is the subject of this review. ZFNs, consisting of DNA binding and nuclease domains, can be designed to recognize specific DNA sequences and thereby enable targeted cleavage (Urnov *et al.* 2010). Alternative nucleases, such as meganucleases (Stoddard 2011), TALENs (Bogdanove and Voytas 2011), and CRISPR/Cas (Shan *et al.* 2013), have also been used to generate targeted DNA breaks. The ability to cleave-specific DNA sequences and promote different mechanisms of DNA repair enable various types of genomic modifications ranging from single-nucleotide mutations to large sequence deletions, rearrangements and/or integrations (Curtin *et al.* 2012).

Although the creation of targeted DNA modifications is a powerful capability, genome editing requires other supporting technologies—not the least of which is the ability to generate, isolate, and propagate modified genomes. It is here that *in vitro* biology plays a particularly critical role. Genome editing reagents, such as designed nucleases and donor template DNA, need to be effectively delivered to target cells, and rare genetic variants must be isolated and captured as ‘events’ for propagation and maintenance. Typically, these operations take advantage of cell and tissue culture methods whereby plant transformation, *in vitro* selection, and regeneration are employed.

The potential value of genome editing technology to basic and applied plant science cannot be overestimated. The ability to modify genetic information in a precise and specific manner and recover modified plants enables not only studies of gene function and biological mechanisms, but also, potentially, the creation of novel phenotypes. As the demand for agricultural output grows with an expanding human population, the need to engineer more complex traits in crops, such as enhanced yield and stress tolerance, will require more sophisticated approaches. Genome editing *via* designed nucleases represents one of the critical enabling capabilities for future crop improvement.

## Designed ZFNs

**Figure 1.**
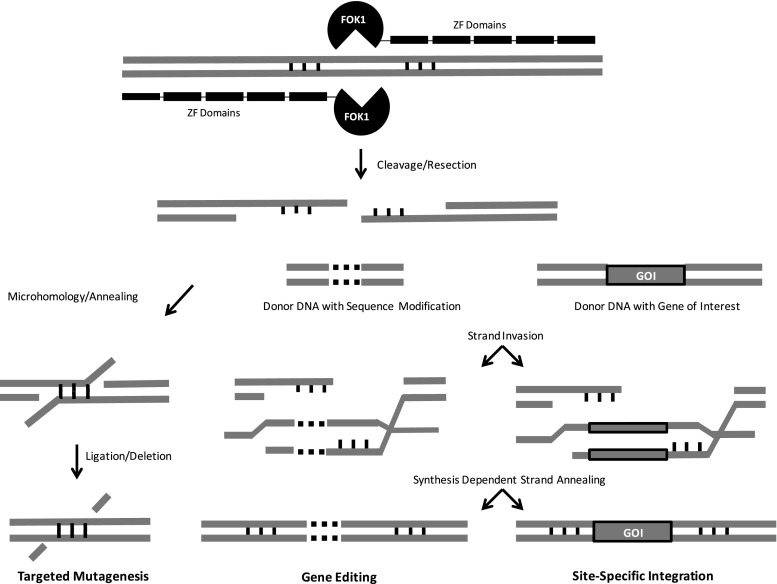
Targeted genome modification via double-strand break (DSB) repair. Zinc finger nucleases (ZFNs) bind to a target sequence, thereby dimerizing *Fok*I nuclease. The DSB generated by ZFN cleavage induces DNA repair processes. In the absence of donor template DNA, error-prone non-homologous end joining (NHEJ) can result in ‘targeted mutagenesis’ (*left*). In the presence of homologous sequences, homology-directed repair (HDR) can result in ‘gene editing’ (*center*) or ‘site-specific integration’ (*right*).

ZFNs consist of zinc finger protein domains, capable of sequence-specific DNA binding, fused to a nuclease domain for DNA cleavage (Fig. 1). DNA binding is the result of a tethered array of 4–6 zinc finger protein domains that each recognize approximately 3 bp of DNA. Although more-or-less modular with respect to binding specificity, there are considerable context effects, i.e., interactions with neighboring domains, which make their binding more specific but their design somewhat challenging (Urnov *et al.* 2010). They are most effectively assembled from an archive of two-finger modules that each recognize specific 6-bp DNA sequences whereby domain junctions within each module are optimized for sequence recognition (Moore *et al.* 2001). For DNA cleavage, the catalytic domain of the type II restriction enzyme *Fok*I has been used (Kim *et al.* 1996). A critical property of the catalytic domain is that it must dimerize to cleave DNA, so two adjacent ZFN pairs must orient themselves with appropriate spacing at the target site (Fig. 1). Although a somewhat larger gene product needs to be expressed, the longer recognition sequences (24–36 bp) required for binding result in a higher level of specificity. In addition, *Fok*I variants requiring heterodimerization have been developed, thereby further enhancing sequence specificity and reducing off-site cleavage (Miller *et al.* 2007).

ZFNs can be designed to bind and cleave virtually any stretch of DNA sequence, thereby allowing for the creation of DNA double-strand breaks (DSBs) at specified loci. Expression of genes encoding ZFNs and concomitant cleavage at endogenous genomic loci has been demonstrated in a broad spectrum of organisms, including human (Urnov *et al.* 2005; Lombardo *et al.* 2007; Moehle *et al.* 2007; Perez *et al.* 2008; Sebastiano *et al.* 2011; Wilen *et al.* 2011; Provasi *et al.* 2012), hamster (Santiago *et al.* 2008), mouse (Osiak *et al.* 2011), pig (Hauschild *et al.* 2011), frog (Young *et al.* 2011), zebra fish (Doyon *et al.* 2008), insect (Beumer *et al.* 2006; Bibikova *et al.* 2002), roundworm (Morton *et al.* 2006), and plasmodium (Straimer *et al.* 2012). The present review focuses on the use of ZFNs for cleaving genomic loci in plants.

## Repairing DSBs

A cell’s capacity to create and repair DSBs is central to the facilitation of recombination between DNA sequences—so essential to both the maintenance of genomic integrity and the generation of genetic variability. DSB repair can occur using homologous sequences as templates for synthesis (Moynahan and Jasin 2010). Such homology-directed repair (HDR) uses sister chromatids, homologous chromosomes, or other related DNA. Alternative repair pathways involve non-homologous end joining (NHEJ) of broken ends (Lieber 2010). Targeted sequence modification (i.e., genome editing) is predicated on the ability to create sequence-specific DSBs and exploit the cell’s DNA repair machinery to generate desired genetic outcomes.

The genetic outcome of the resolution of a DSB generated by ZFN cleavage is a function of the process used to repair it (Fig. 1). In plants, the most common DNA repair mechanism appears to involve NHEJ, where broken ends are simply religated (Puchta 2005). This repair process can be error-prone, resulting in small insertions, deletions, and/or rearrangements (Gorbunova and Levy 1999). If imprecise DSB repair occurs in a gene sequence, mutations can be introduced which may affect function. Indeed, this represents a general method of targeted mutagenesis whereby ZFNs designed to bind and cleave a specific genomic locus can be introduced and expressed, resulting in NHEJ-mediated sequence alteration (Fig. 1).

In the presence of DNA sequences homologous to those flanking the DSB, HDR can use such sequences as templates for synthesis-dependent strand annealing (Haber 2000). If the homologous sequences are investigator-designed donor DNA, the resolution of a DSB can be dictated by the composition of the repair template. In other words, sequence modifications can be copied into the targeted cleavage site and can range from single- to few-base-pair modifications (i.e., gene editing) to the integration of complete transgene expression cassettes (i.e., site-specific integration; Fig. 1).

## ZFN-Mediated Targeted Mutagenesis

Mutation breeding dates back to the 1920s with the discovery of the mutagenic effect of X-rays on plant genes (Stadler 1928). Since then, a wide range of characters including plant architecture, flowering, and maturity have been modified through mutation breeding, and over 3000 novel cultivars have been developed and deployed in agriculture (Mba 2013). Historically, ‘forward genetic’ approaches have been used whereby direct screening for specific phenotypes has resulted in germplasm with enhanced performance for use in breeding. These approaches are limited by the ability to generate, identify, and isolate rare individuals and the fact that, since most mutations are recessive, gene redundancies tend to mask any phenotypic effects accompanying a given sequence modification. More recently, ‘reverse genetic’ approaches have become possible whereby sequence modifications of specific genes are identified using high-throughput genome analysis (Chen *et al.* 2014). Difficulties with these approaches include the lack of understanding of which genes to mutate to generate useful phenotypes and the random nature of the sequence modifications induced by physical or chemical means. The use of designed nucleases to target specific modifications of selected genome sequences adds a level of control to this process and represents a significant improvement of the ‘reverse genetic’ approach.

### Sequence modification of preintegrated reporter constructs.

Early proof-of-concept studies of ZFN-mediated targeted mutagenesis in plants involved modification of preintegrated sequences comprising ZFN cleavage sites. One such study involved the expression of a ZFN under the control of a heat-shock protein promoter whereby an *Eco*RI restriction site could be lost upon mutation (Lloyd *et al.* 2005). Mutation frequencies as high as 19.6% were observed in heat-treated Arabidopsis seedlings, and sequencing revealed that most mutations were simple deletions of 1–52 bp. In a similar study, constitutive expression of a ZFN in stably transformed Arabidopsis resulted in a 2% mutation frequency and deletions ranging from 1 to 80 bp (de Pater *et al.* 2009). A preintegrated reporter gene containing a ZFN cleavage site has also been used to study targeted mutagenesis. A gene encoding the enzyme β-glucuronidase (*GUS*) with a stop codon within a ZFN cleavage site was stably integrated into Arabidopsis, and subsequent co-cultivation with an *Agrobacterium* strain harboring a corresponding ZFN expression cassette resulted in sectors of GUS staining (Tovkach *et al.* 2009). Sequence analysis of the target site revealed several single-nucleotide deletions and substitutions in the stop codon, resulting in a *GUS* open reading frame. Similar results were obtained when the ZFN was expressed using a viral vector (Vainstein *et al.* 2011).

### *Sequence modification of endogenous genes.*

Endogenous genomic loci have also been mutated following ZFN expression. Genes encoding ZFNs designed to cleave within the *ABA-INSENSITIVE-4 (ABI4)* gene, driven by a heat-shock protein promoter, were stably integrated into Arabidopsis (Osakabe *et al.* 2010). After heat induction, mutations in *ABI4* were observed in somatic cells at frequencies up to 3%, and homozygous mutant T_3_ progeny displayed the expected loss-of-function phenotype for this gene, i.e., ABA and glucose insensitivity. In a similar study, genes encoding ZFNs that recognize the Arabidopsis *ALCOHOL DEHYDROGENASE-1 (ADH1)* and *TRANSPARENT TESTA-4 (TT4)* genes were expressed in Arabidopsis under the control of an estrogen-inducible promoter (Zhang *et al.* 2010). Induced T_1_ plants exhibited somatic mutation frequencies of 7 and 16% for *ADH1* and *TT4*, respectively, and the mutations ranged from insertions of 1–2 bp to deletions of 3–142 bp. No evidence of ZFN-induced ‘off-target’ cleavage was observed following PCR amplification and sequencing of ectopic Arabidopsis genomic sequences most similar to *ADH1* and *TT4*. Mutations were transmitted to progeny at frequencies of 69 and 33% for *ADH1* and *TT4*, respectively, and 20% appeared to be homozygous, suggesting biallelic mutation. In soybean, a gene encoding a ZFN targeting two paralogous *DICER-LIKE (DCL4b)* genes, *DCL4a* and *DCL4b*, under the control of an estrogen-inducible promoter, was delivered using *Agrobacterium* in the presence of estrogen (Curtin *et al.* 2011). Three T_0_ plants were recovered, and sequence analysis of PCR-amplified products revealed that one of the plants had an adenine-base insertion at the *DCL4a* locus and another had a two-base thymine and adenine insertion into *DCL4b*. Both plants appeared to be heterozygous for the mutation. The plant with the *dcl4a* mutation exhibited phenotypic abnormalities, including aborted seed. The *dcl4b* plant appeared normal and produced T_1_ progeny in which the *dcl4b* mutation segregated 1:2:1 as expected. These results provide clear evidence that expression of genes encoding ZFNs can generate heritable mutations at targeted endogenous loci.

### *Gene deletion.*

**Figure 2.**
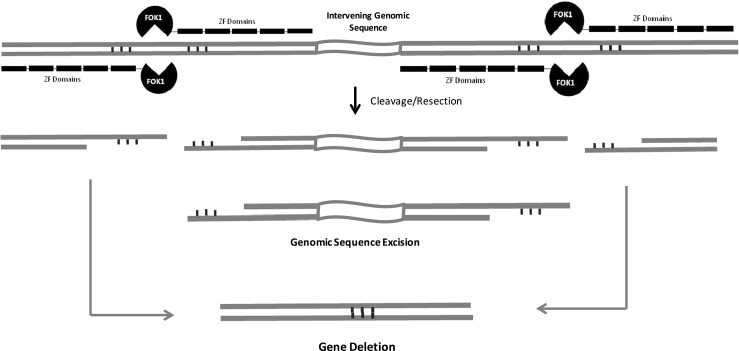
Gene deletion. Two concurrent zinc finger nuclease (ZFN)-mediated double-strand breaks (DSBs) can result in the loss of intervening sequences.

In addition to small, NHEJ-induced mutations, ZFN-mediated cleavage can result in larger DNA sequence deletions (Fig. 2). A reporter construct containing a tandem repeat of 540 bp of partial, i.e., 3′/5′, *GREEN FLUORESCENT PROTEIN (GFP)* gene fragments with 2.8 kb of intervening heterologous DNA sequence containing a ZFN cleavage site was stably integrated into tobacco (Cai *et al.* 2009). Subsequent expression of the corresponding ZFN gene resulted in targeted DSB formation, recombination between the *GFP* gene fragments, and deletion of the intervening 2.8-kb sequence. Deletion of a 4.3-kb integrated *GUS* gene sequence flanked by ZFN cleavage sites was observed in 35% of the F_1_ progenies when crossed to a ZFN-expressing plant (Petolino *et al.* 2010). Even larger deletions (e.g., 55 kb) were observed following nuclease cleavage within tandem gene clusters (Voytas 2013). These results provide proof of concept for a potentially powerful means of creating targeted genome sequence deletions.

### *Gene editing using HDR.*

A more precise means of generating targeted mutations at specific genomic locations involves the use of donor template DNA sequences to facilitate HDR of DSBs. Typically, donor template DNA for HDR contains approximately 750- to 1000-kb stretches of sequence homologous to that flanking the genomic cleavage site and the desired sequence modification. Unfortunately, the frequency of HDR in somatic plant cells appears to be extremely low, so the identification and isolation of such modifications can be a challenge. Nonetheless, selectable phenotypes have been used to demonstrate gene editing *via* HDR. Specific mutations to the *SULFONYLUREA RECEPTOR* genes *SuRA* and *SuRB* in tobacco confer resistance to certain acetolactate synthase (ALS)-inhibiting herbicides. Herbicide-resistant mutants were isolated following co-introduction of genes encoding designed ZFNs and donor template DNA into tobacco protoplasts (Townsend *et al.* 2009). Mutation frequencies exceeding 2% were observed and mutations as far as 1.3 kb from the ZFN cleavage site were obtained. The Arabidopsis *PROTOPORPHYRINOGEN OXIDASE (PPOX)* gene has also been edited using HDR of a ZFN-mediated DSB (de Pater *et al.* 2013). In this case, two mutations resulting in resistance to the herbicide butafenacil were delivered on a donor template DNA, and an editing frequency of 3.1 × 10^−3^ was observed and transmitted to the next generation. One of the future challenges for gene editing *via* HDR will involve the targeted modification of endogenous genes without selectable phenotypes. This may necessitate the use of high-throughput analyses to screen for the desired outcome.

## Targeted Transgene Integration

Integrating DNA sequences into predetermined genomic locations would greatly enhance the precision of plant transformation and predictability of transgenic plant performance. Random DNA integration carries not only the risk of potential unwanted insertional mutations, but also the unpredictable consequences of position effect on transgene behavior. In addition, transgene stacking *via* sequential random transformation requires resource-intensive introgression *via* conventional breeding. Targeting DNA sequences to specified genomic loci allows for sequential transgene stacking into a single locus, thereby simplifying downstream breeding (Ainley *et al.* 2013).

### *Targeted integration into preintegrated loci.*

The initial demonstrations of ZFN-mediated, site-specific transgene integration involved correcting preintegrated selectable marker genes (Wright *et al.* 2005; Cai *et al.* 2009). In one study using tobacco protoplasts, a defective fusion between genes encoding the GUS reporter and neomycin phosphotransferase (NPTII) selectable marker proteins was corrected *via* co-delivery of a gene encoding a ZFN, which cleaved in the junction between the two genes, and a donor template DNA comprising sequences homologous to those flanking the ZFN cleavage site and the missing *GUS/NPTII* gene sequences (Wright *et al.* 2005). HDR occurred in more than 10% of the protoplasts as estimated by positive *GUS* expression. Targeting in this system was enhanced more than two orders of magnitude when the ZFN and donor template DNA were expressed from a geminivirus (Baltes *et al.* 2014). In a similar study, a preintegrated construct containing a 3′ partial *PHOSPHINOTHRICIN ACETYL TRANSFERASE (PAT)* gene fragment flanked by a ZFN binding site and an Arabidopsis *4-COUMARYL SYNTHASE (4-CoAS)* intron allowed for *in vitro* selection on Herbiace®-containing medium following ZFN-mediated cleavage and targeted integration of a complementary 5′ *PAT* gene fragment with a homologous *4-CoAS* intron delivered *via Agrobacterium* (Cai *et al.* 2009). Preintegrated loci have also been successfully targeted using NHEJ (Tzfira *et al.* 2012). In this system, identical ZFN cleavage sites were present in the genome and on the donor template DNA. Co-delivery of a ZFN expression construct and donor template DNA resulted in targeted DSB formation at the preintegrated locus and linearization and subsequent targeted integration of the donor template DNA *via* NHEJ (Weinthal *et al.* 2013).

### *Targeted integration into endogenous loci.*

ZFNs designed to cleave endogenous genomic sequences have been used to facilitate site-specific transgene integration into native genes. A gene encoding a ZFN designed to cleave a tobacco *ENDOCHITINASE-50 (CHN50)* gene sequence was co-delivered *via Agrobacterium* along with a *PAT* herbicide-resistance gene flanked on each side by 750 bp of *CHN50* gene sequence (Cai *et al.* 2009). The majority of transgenic events were the result of random integration; however, 5–10% of the events appeared to have targeted the *CHN50* locus. In a similar study, genes encoding ZFNs designed to cleave in exon 2 of the maize *INOSITOL-1,3,4,5,6-PENTAKISPHOSPHATE KINASE 1 (IPK1)* gene were co-delivered with donor template DNA containing a promoterless *PAT* gene with a 2A ‘stutter’ sequence flanked by 815 bp of sequence homologous to *IPK1* (Shukla *et al.* 2009). Targeted cleavage at the *IPK1* locus and precise ‘trapping’ of the *IPK1* promoter resulted in site-specific integration and herbicide resistance.

## Targeting in DNA Repair Mutants

The genetic outcome of the resolution of a DSB is largely a function of the pathway used for DNA repair, i.e., HDR versus NHEJ. In an attempt to direct the resolution of repair and enhance mutation and/or targeting frequencies, genes encoding ZFNs have been delivered into various genetic backgrounds in which specific genes involved in DNA repair have been mutated. The frequency of ZFN-mediated deletions larger than 4 bp in the Arabidopsis *ABI4* gene was 2.6-fold greater in a genetic background deficient in KU80, an enzyme involved in NHEJ DNA repair (Osakabe *et al.* 2010). Similarly, although the frequency of ZFN-mediated mutagenesis at the endogenous *ADH1* locus in Arabidopsis was not increased in genetic backgrounds with mutations in genes encoding KU70 or LIG4, two other DNA repair proteins, DNA repair appeared to be shifted to microhomology-dependent NHEJ, resulting in larger deletions than in a wild-type background (Qi *et al.* 2013). In addition, *ADH1* mutagenesis increased up to eightfold in a genetic background with a mutated gene encoding SMC6B, a protein involved in DSB repair using sister chromatids. Targeted sequence integration into the *ADH1* locus *via* HDR increased 16-fold and 4-fold, respectively, in genetic backgrounds with mutations in genes encoding KU70 and LIG4 (Qi *et al.* 2013). Using a different approach to DNA repair pathway manipulation, coexpression of genes encoding a ZFN and RAD54, an enzyme involved in HDR in yeast, both driven by an egg cell-specific promoter, resulted in a tenfold increase in gene targeting in Arabidopsis (Even-Faitelson *et al.* 2011). These results suggest that, although it adds a level of complexity to the overall genome editing process, manipulating DNA repair pathways provides a level of control over the types of sequence modifications resulting from ZFN-mediated cleavage and a potential path to increased mutagenesis and gene targeting frequencies.

## *In Vitro* Biology and Genome Editing

The first prerequisite to ZFN-mediated genome editing is the ability to effectively deliver macromolecules such as DNA, RNA, and/or protein to cells to facilitate DNA sequence modification. Standard plant transformation methods such as polyethylene glycol delivery into protoplasts (Wright *et al.* 2005; Townsend *et al.* 2009), microparticle bombardment (Ainley *et al.* 2013), WHISKERS™ (Shukla *et al.* 2009), and *Agrobacterium* (Cai *et al.* 2009; de Pater *et al.* 2009, 2013) have been successfully used to deliver such reagents for genome editing. Most recently, genome editing reagents have been successfully delivered to plant cells using non-integrating viruses (Vainstein *et al.* 2011; Baltes *et al.* 2014). The goal of any delivery system is to assure that active ZFNs and, if necessary, donor template DNA is present at the appropriate stage of the cell cycle to enable the targeted cleavage and subsequent DNA repair.

In addition to making specific DNA sequence modifications, it is critical to capture the genetic change in a transmissible form, e.g., in meristems and germlines. Since genetic modification is fundamentally a cellular phenomenon, being able to grow complex organisms such as higher plants as cultured cells and tissues allows for the isolation of clonal variants. Indeed, as with conventional transgenic production, the development and deployment of *in vitro* methods for plant species is equally important to genome editing. In addition, the ability to induce tissue proliferation (e.g., callus) and regeneration of modified plants enables the performance of various compositional and functional analyses critical for both genome editing and capture of genetic variants for potential breeding applications. It is no coincidence, therefore, that the efficacy of genome editing technology for a given species is directly proportional to the ability to perform the full array of somatic cell genetics techniques.

In an attempt to circumvent the need for robust plant cell and tissue culture systems, *in planta* gene targeting has been attempted using the meganuclease I-*Sce*I (Fauser *et al.* 2012; Ayar *et al.* 2013). This involved the preintegration of target and donor template DNA and the expression of a genes encoding I-*Sce*I to facilitate cleavage and intrachromosomal DNA repair. In Arabidopsis, the expression of a gene encoding I-*Sce*I resulted in the concurrent cleavage of preintegrated sequences flanking donor template DNA comprising a promoterless *GUS* reporter gene and a genomic target sequence with a corresponding nuclease cleavage site (Fauser *et al.* 2012). The preintegrated donor template was excised and used to repair the DSB at the target site, resulting in *GUS* expression. Roughly 1% of the progenies from *GUS*-expressing plants were shown to be targeted. The authors concluded that this method could avoid the need for high-frequency plant transformation and be particularly valuable in more recalcitrant species. However, a similar study in maize required *in vitro* selection and plant regeneration to isolate targeted progeny (Ayar *et al.* 2013). In this case, a gene encoding an NPTII selectable marker was functionalized and kanamycin selection was used to identify and capture targeted events.

Genome editing for the development of complex traits is expected to involve the need to deliver many experimental constructs into cells and tissues and to analyze numerous genetic variants. Automated cell and tissue handling opens up the possibility for such high-throughput manipulations (Knoll *et al.* 2004) but may require growing protoplasts or cell suspension cultures in small volumes in a multi-well format. In addition, phenotyping complex traits such as stress tolerance or yield will require the recovery of modified plants. Should genome editing move in this direction, the need for robust *in vitro* cell and tissue culture systems will be even more evident.

## Conclusions and Future Prospects

The use of ZFNs and other designed nucleases to make targeted DSBs has opened up the possibility of precision genome editing. It is anticipated that genome editing will have a major impact on at least two broad areas of plant biology. The first is functional genomics, whereby the ability to make precise DNA sequence modifications to targeted endogenous loci will advance basic understanding of genome structure and function as well as the mechanisms that give rise to phenotypes. Such enhanced understanding should lead to more effective hypothesis-driven gene discovery efforts for novel trait development. The second area where genome editing technology will have a major impact is in applied crop improvement and commercial product development. ‘Reverse genetic’ approaches to mutation breeding will no doubt be revolutionized by virtue of being able to make targeted DNA sequence modifications rather than random changes. Products developed from such non-transgenic enhancements are likely to require an altered path to deregulation, i.e., similar to conventionally bred genotypes, and thus be more cost-effective to bring to market. In addition, current transgenic production technology and the random insertion of DNA sequences involve the need to generate and screen numerous unproductive events. Targeting transgenes to predetermined genomic loci will not only reduce undesired side-effects and increase the predictability of transgene performance but will also simplify event characterization and allow for sequential transgene stacking, thereby reducing product development time and cost.

Clearly, genome editing shows great promise for both basic and applied plant science. For this promise to be met, supporting technologies such as high resolution molecular methods for genome analysis and bioinformatics for trait characterization are required. However, no supporting technology is more critical than the ability to create and isolate genetic variants. As such, *in vitro* manipulation of plant cells and tissues will continue to play a central role in the further development of genome editing technology.

## References

[CR1] Ainley WM, Sastry-Dent L, Welter ME, Murray MG, Zeitler B, Amora R, Corbin DR, Miles RR, Arnold NL, Strange TL, Simpson MA, Cao Z, Carroll C, Pawelczak KS, Blue R, West K, Rowland LM, Perkins D, Samuel P, Dewes CM, Shen L, Sriram S, Evans SL, Rebar EJ, Zhang L, Gregory PD, Urnov FD, Webb SR, Petolino JF (2013). Trait stacking via targeted genome editing. Plant Biotechnol J.

[CR2] Ayar A, Wehrkamp-Richter S, Laffaire JB, Le Goff S, Levy J, Chaignon S, Salmi H, Lepicard A, Sallaud C, Gallego ME, White CI, Paul W (2013). Gene targeting in maize by somatic ectopic recombination. Plant Biotechnol J.

[CR3] Baltes NJ, Gil-Humanes J, Cermak T, Atkins PA, Voytas DF (2014). DNA replicons for plant genome engineering. Plant Cell.

[CR4] Beumer K, Bhattacharyya G, Bibikova M, Trautman JK, Carroll D (2006). Efficient gene targeting in drosophila with zinc-finger nucleases. Genetics.

[CR5] Bibikova M, Golic M, Golic KG, Carroll D (2002). Targeted chromosomal cleavage and mutagenesis in drosophila using zinc-finger nucleases. Genetics.

[CR6] Bogdanove AJ, Voytas DF (2011). TAL effectors: Customizable proteins for DNA targeting. Science.

[CR7] Cai CQ, Doyon Y, Ainley WM, Miller JC, Dekelver RC, Moehle EA, Rock JM, Lee YL, Garrison R, Schulenberg L, Blue R, Worden A, Baker L, Faraji F, Zhang L, Holmes MC, Rebar EJ, Collingwood TN, Rubin-Wilson B, Gregory PD, Urnov FD, Petolino JF (2009). Targeted transgene integration in plant cells using designed zinc finger nucleases. Plant Mol Biol.

[CR8] Chen L, Hao L, Parry MAJ, Phillips AL, Hu Y-G (2014). Progress in TILLING as a tool for functional genomics and improvement of crops. J Integr Plant Biol.

[CR9] Curtin SJ, Voytas DF, Stupar RM (2012). Genome engineering of crops with designer nucleases. Plant Genome.

[CR10] Curtin SJ, Zhang F, Sander JD, Haun WJ, Starker C, Baltes NJ, Reyon D, Dahlborg EJ, Goodwin MJ, Coffman AP, Dobbs D, Joung JK, Voytas DF, Stupar RM (2011). Targeted mutagenesis of duplicated genes in soybean with zinc-finger nucleases. Plant Physiol.

[CR11] de Pater S, Neuteboom LW, Pinas JE, Hooykaas PJ, van der Zaal BJ (2009). ZFN-induced mutagenesis and gene-targeting in Arabidopsis through *Agrobacterium*-mediated floral dip transformation. Plant Biotechnol J.

[CR12] de Pater S, Pinas JE, Hooykaas PJ, van der Zaal BJ (2013). ZFN-mediated gene targeting of the Arabidopsis protoporphyrinogen oxidase gene through *Agrobacterium*-mediated floral dip transformation. Plant Biotechnol J.

[CR13] Doyon Y, McCammon JM, Miller JC, Faraji F, Ngo C, Katibah GE, Amora R, Hocking TD, Zhang L, Rebar EJ, Gregory PD, Urnov FD, Amacher SL (2008). Heritable targeted gene disruption in zebrafish using designed zinc-finger nucleases. Nat Biotechnol.

[CR14] Even-Faitelson L, Samach A, Melamed-Bessudo C, Avivi-Ragolsky N, Levy AA (2011). Localized egg-cell expression of effector proteins for targeted modification of the Arabidopsis genome. Plant J.

[CR15] Fauser F, Roth N, Pacher M, Ilg G, Sanchez-Fernandez R, Biesgen C, Puchta H (2012). *In planta* gene targeting. Proc Natl Acad Sci U S A.

[CR16] Gorbunova V, Levy AA (1999). How plants make ends meet: DNA double-strand break repair. Trends Plant Sci.

[CR17] Haber JE (2000). Partners and pathways: repairing a double-strand break. Trends Genet.

[CR18] Hauschild J, Petersen B, Santiago Y, Queisser A-L, Carnwath JW, Lucas-Hahn A, Zhang L, Meng X, Gregory PD, Schwinzer R, Cost GJ, Niemann H (2011). Efficient generation of a biallelic knockout in pigs using zinc-finger nucleases. Proc Natl Acad Sci U S A.

[CR19] Kim YG, Cha J, Chandrasegaran S (1996). Hybrid restriction enzymes: Zinc finger fusions to *Fok* I cleavage domain. Proc Natl Acad Sci U S A.

[CR20] Knoll A, Scherer T, Poggendorf I, Lütkemeyer D, Lehmann J (2004). Flexible automation of cell culture and tissue engineering tasks. Biotechnol Prog.

[CR21] Lieber MR (2010). The mechanism of double-strand DNA break repair by the nonhomologous DNA end-joining pathway. Annu Rev Biochem.

[CR22] Lloyd A, Plaisier CL, Carroll D, Drews GN (2005). Targeted mutagenesis using zinc-finger nucleases in Arabidopsis. Proc Natl Acad Sci U S A.

[CR23] Lombardo A, Genovese P, Beausejour CM, Colleoni S, Lee YL, Kim KA, Ando D, Urnov FD, Galli C, Gregory PD, Holmes MC, Naldini L (2007). Gene editing in human stem cells using zinc finger nucleases and integrase-defective lentiviral vector delivery. Nat Biotechnol.

[CR24] Mba C (2013). Induced mutations unleash the potentials of plant genetic resources for food and agriculture. J. Agron.

[CR25] Michael TP, Jackson S (2013) The first 50 plant genomes. Plant Genome 6. doi:10.3835/plantgenome2013.03.0001in

[CR26] Miller JC, Holmes MC, Wang J, Guschin DY, Lee YL, Rupniewski I, Beausejour CM, Waite AJ, Wang NS, Kim KA, Gregory PD, Pabo CO, Rebar EJ (2007). An improved zinc-finger nuclease architecture for highly specific genome editing. Nat Biotechnol.

[CR27] Moehle EA, Rock JM, Lee YL, Jouvenot Y, DeKelver RC, Gregory PD, Urnov FD, Holmes MC (2007). Targeted gene addition into a specified location in the human genome using designed zinc finger nucleases. Proc Natl Acad Sci U S A.

[CR28] Moore M, Klug A, Choo Y (2001). Improved DNA binding specificity from polyzinc finger peptides by using strings of two-finger units. Proc Natl Acad Sci U S A.

[CR29] Morton J, Davis MW, Jorgensen EM, Carroll D (2006). Induction and repair of zinc-finger nuclease-targeted double-strand breaks in *Caenorhabditis elegans* somatic cells. Proc Natl Acad Sci U S A.

[CR30] Moynahan ME, Jasin M (2010). Mitotic homologous recombination maintains genomic stability and suppresses tumorigenesis. Nat Rev Mol Cell Biol.

[CR31] Osakabe K, Osakabe Y, Toki S (2010). Site-directed mutagenesis in Arabidopsis using custom-designed zinc finger nucleases. Proc Natl Acad Sci U S A.

[CR32] Osiak A, Radecke F, Guhl E, Radecke S, Dannemann N, Lütge F, Glage S, Rudolph C, Cantz T, Schwarz K, Heilbronn R, Cathomen T (2011). Selection-independent generation of gene knockout mouse embryonic stem cells using zinc-finger nucleases. PLoS One.

[CR33] Perez EE, Wang J, Miller JC, Jouvenot Y, Kim KA, Liu O, Wang N, Lee G, Bartsevich VV, Lee YL, Guschin DY, Rupniewski I, Waite AJ, Carpenito C, Carroll RG, Orange JS, Urnov FD, Rebar EJ, Ando D, Gregory PD, Riley JL, Holmes MC, June CH (2008). Establishment of HIV-1 resistance in CD4^+^ T cells by genome editing using zinc-finger nucleases. Nat Biotechnol.

[CR34] Petolino JF, Worden A, Curlee K, Connell J, Strange Moynahan TL, Larsen C, Russell S (2010). Zinc finger nuclease-mediated transgene deletion. Plant Mol Biol.

[CR35] Provasi E, Genovese P, Lombardo A, Magnani Z, Liu P-Q, Reik A, Chu V, Paschon DE, Zhang L, Kuball J, Camisa B, Bondanza A, Casorati G, Ponzoni M, Ciceri F, Bordignon C, Greenberg PD, Holmes MC, Gregory PD, Naldini L, Bonini C (2012). Editing T cell specificity towards leukemia by zinc finger nucleases and lentiviral gene transfer. Nat Med.

[CR36] Puchta H (2005). The repair of double-strand breaks in plants: mechanisms and consequences for genome evolution. J Exp Bot.

[CR37] Qi Y, Zhang Y, Zhang F, Baller JA, Cleland SC, Ryu Y, Starker CG, Voytas DF (2013). Increasing frequencies of site-specific mutagenesis and gene targeting in Arabidopsis by manipulating DNA repair pathways. Genome Res.

[CR38] Santiago Y, Chan E, Liu PQ, Orlando S, Zhang L, Urnov FD, Holmes MC, Guschin D, Waite A, Miller JC, Rebar EJ, Gregory PD, Klug A, Collingwood TN (2008). Targeted gene knockout in mammalian cells by using engineered zinc-finger nucleases. Proc Natl Acad Sci U S A.

[CR39] Sebastiano V, Maeder ML, Angstman JF, Haddad B, Khayter C, Yeo DT, Goodwin MJ, Hawkins JS, Ramirez CL, Batista LFZ, Artandi SE, Wernig M, Joung JK (2011). *In situ* genetic correction of the sickle cell anemia mutation in human induced pluripotent stem cells using engineered zinc finger nucleases. Stem Cells.

[CR40] Shan Q, Wang Y, Li J, Zhang Y, Chen K, Liang Z, Zhang K, Liu J, Xi JJ, Qiu JL, Gao C (2013). Targeted genome modification of crop plants using a CRISPR-Cas system. Nat Biotechnol.

[CR41] Shukla VK, Doyon Y, Miller JC, DeKelver RC, Moehle EA, Worden SE, Mitchell JC, Arnold NL, Gopalan S, Meng X, Choi VM, Rock JM, Wu YY, Katibah GE, Zhifang G, McCaskill D, Simpson MA, Blakeslee B, Greenwalt SA, Butler HJ, Hinkley SJ, Zhang L, Rebar EJ, Gregory PD, Urnov FD (2009). Precise genome modification in the crop species *Zea mays* using zinc-finger nucleases. Nature.

[CR42] Stadler LJ (1928). Genetic effects of X-rays in maize. Proc Natl Acad Sci U S A.

[CR43] Stoddard BL (2011). Homing endonucleases: from microbial genetic invaders to reagents for targeted DNA modification. Structure.

[CR44] Straimer J, Lee MCS, Lee AH, Zeitler B, Williams AE, Pearl JR, Zhang L, Rebar EJ, Gregory PD, Llinas M, Urnov FD, Fidock DA (2012). Site-specific genome editing in *Plasmodium falciparum* using engineered zinc-finger nucleases. Nat Methods.

[CR45] Tovkach A, Zeevi V, Tzfira T (2009). A toolbox and procedural notes for characterizing novel zinc finger nucleases for genome editing in plant cells. Plant J.

[CR46] Townsend JA, Wright DA, Winfrey RJ, Fu F, Maeder ML, Joung JK, Voytas DF (2009). High-frequency modification of plant genes using engineered zinc-finger nucleases. Nature.

[CR47] Tzfira T, Weinthal D, Marton I, Zeevi V, Zuker A, Vainstein A (2012). Genome modifications in plant cells by custom-made restriction enzymes. Plant Biotechnol J.

[CR48] Urnov FD, Miller JC, Lee YL, Beausejour CM, Rock JM, Augustus S, Jamieson AC, Porteus MH, Gregory PD, Holmes MC (2005). Highly efficient endogenous human gene correction using designed zinc-finger nucleases. Nature.

[CR49] Urnov FD, Rebar EJ, Holmes MC, Zhang HS, Gregory PD (2010). Genome editing with engineered zinc finger nucleases. Nat Rev Genet.

[CR50] Vainstein A, Marton I, Zuker A, Danziger M, Tzfira T (2011). Permanent genome modifications in plant cells by transient viral vectors. Trends Biotechnol.

[CR51] Voytas DF (2013). Plant genome engineering with sequence-specific nucleases. Annu Rev Plant Biol.

[CR52] Weinthal DM, Taylor RA, Tzfira T (2013). Nonhomologous end joining-mediated gene replacement in plant cells. Plant Physiol.

[CR53] Wilen CB, Wang J, Tilton JC, Miller JC, Kim KA, Rebar EJ, Sherrill-Mix SA, Patro SC, Secreto AJ, Jordan APO, Lee G, Kahn J, Aye PP, Bunnell BA, Lackner AA, Hoxie JA, Danet-Desnoyers GA, Bushman FD, Riley JL, Gregory PD, June CH, Holmes MC, Doms RW (2011). Engineering HIV-resistant human CD4+ T cells with CXCR4-specific zinc-finger nucleases. PLoS Pathog.

[CR54] Wingender E, Chen X, Hehl R, Karas H, Liebich I, Matys V, Meinhardt T, Pruss M, Reuter I, Schacherer F (2000). TRANSFAC: an integrated system for gene expression regulation. Nucleic Acids Res.

[CR55] Wright DA, Townsend JA, Winfrey RJ, Irwin PA, Rajagopal J, Lonosky PM, Hall BD, Jondle MD, Voytas DF (2005). High-frequency homologous recombination in plants mediated by zinc-finger nucleases. Plant J.

[CR56] Young JJ, Cherone JM, Doyon Y, Ankoudinova I, Faraji FM, Lee AH, Ngo C, Guschin DY, Paschon DE, Miller JC, Zhang L, Rebar EJ, Gregory PD, Urnov FD, Harland RM, Zeitler B (2011). Efficient targeted gene disruption in the soma and germ line of the frog *Xenopus tropicalis* using engineered zinc-finger nucleases. Proc Natl Acad Sci U S A.

[CR57] Zhang F, Maeder ML, Unger-Wallace E, Hoshaw JP, Reyon D, Christian M, Li X, Pierick CJ, Dobbs D, Peterson T, Joung JK, Voytas DF (2010). High frequency targeted mutagenesis in *Arabidopsis thaliana* using zinc finger nucleases. Proc Natl Acad Sci U S A.

